# Stress-Induced Depression and Its Effects on Tooth Wear in Rats: A 3D Dental Scan Imaging Perspective

**DOI:** 10.3390/life15050712

**Published:** 2025-04-28

**Authors:** Preeyarat Plongniras, Sarawut Lapmanee, Natchayaporn Thonapan, Phuripong Thangsombat, Phongsakorn Janthaphim, Chanakarn Lertkarnvijai, Pattama Chailertvanitkul, Supawich Morkmued

**Affiliations:** 1 Department of Prosthodontics, Faculty of Dentistry, Khon Kaen University, Khon Kaen 40002, Thailand; preepl@kku.ac.th; 2 Chulabhorn International College of Medicine, Thammasat University, Pathum Thani 12120, Thailand; lapmanee@tu.ac.th; 3 Department of Radiological Technology, Faculty of Allied Health Sciences, Thammasat University, Pathum Thani 12120, Thailand; natchayaporn.t@allied.tu.ac.th; 4 Department of Preventive Dentistry, Pediatric Dentistry Division, Faculty of Dentistry, Khon Kaen University, Khon Kaen 40002, Thailand; 5 Department of Restorative Dentistry, Faculty of Dentistry, Khon Kaen University, Khon Kaen 40002, Thailand; patchai@kku.ac.th

**Keywords:** depression, restraint stress, tooth wear, bruxism, temporomandibular disorder, dental digital scanner, three-dimensional analysis

## Abstract

Background: In addition to behavioral and biochemical abnormalities, a parafunction associated with temporomandibular joint disorders (TMDs) resulted in stress-induced depression in rats. Exploring how chronic stress influences molar wear in rodents provides insights into the understanding of depression, TMD, and oral health. This study aimed to conduct a three-dimensional (3D) analysis of first molar wear in an animal model of depression by comparing molar attrition and cusp variation between stressed male rats and control groups. Methods: After obtaining a validated model of depression in male rats, we obtained 3D scans of lower molars to analyze wear patterns. The 3D analysis was applied to quantify cusps’ volume and the difference in first molar cusp morphological structure. The data were then compared to identify significant morphological differences between groups side by side. Results: The analysis revealed the reduction of cusps’ volume in the depression groups. Rats exposed to depression exhibited significantly greater occlusal table wear than their control counterparts (*p* < 0.05). Conclusions: As dentistry moves towards greater digital imaging, understanding the impact of psychological factors on TMD becomes increasingly necessary. This study shows that stress-induced depression in rats can result in significant tooth wear, as investigated using a 3D dental scanner.

## 1. Introduction

Depression, a condition often associated with adulthood, is increasingly recognized as a significant issue among younger populations. The 2021 National Survey on Drug Use and Health in the USA revealed a concerning trend; out of 21.0 million adults, the prevalence of adults with a major depressive episode was highest among individuals aged 18–25 (18.6%) [1]. Depression also affects approximately 3.8% of the global population, with 5% of adults experiencing the condition, and an estimated 280 million individuals worldwide are living with depression [2]. Additionally, the World Health Organization report further emphasizes the gravity of this issue, ranking depression as the second leading cause of disability worldwide [3].

Biological, psychological, social, and environmental abnormalities are potential factors contributing to the development of depression. In humans, stress levels are associated with muscle asymmetry and non-physiological muscle function. However, the key factor here will be the level of stress [4]. Moreover, chronic stress can activate the hypothalamic–pituitary–adrenal (HPA) axis, resulting in sustained elevations of cortisol in humans and corticosterone in rodents, both of which are commonly referred to as stress hormones. Elevated levels of these stress hormones can have widespread effects on various physiological systems, playing a crucial role in maintaining homeostasis and supporting the proper functioning of multiple organ systems [5]. This demonstrates that their effects extend beyond mental well-being, influencing overall health through various physiological mechanisms.

In depression, the complex interplay between mental and physical health has significant implications for oral health [6,7]. Studies have shown that depression can affect oral tissues, potentially leading to various oral health issues and temporomandibular joint disorders (TMDs) [7,8,9,10]. The findings from research using rat models indicate that depressive states can induce inflammation in the temporomandibular condyle [11], mirroring the TMD observed in human patients. Recently, a meta-regression analysis study showed that TMDs affect 34% of the population, bruxism is less prevalent at 22%, and the co-occurrence of TMDs and bruxism is 17% [12]. This finding underscores the intricate connection between mental health and oral health, suggesting that the effects of depression may manifest in uncertain ways.

Parafunctional habits are characterized by abnormal and repetitive behaviors beyond typical oral functions. The most common manifestations are bruxism and clenching, occurring both during sleep and waking hours. These habits are often associated with tooth wear, altered muscle function, and changes in temporomandibular joint dynamics; however, the exact nature of these relationships remains a subject of ongoing study [13]. While there are no definitive indicators for bruxism in patients, studies have suggested a potential link between this condition and depression, as individuals with bruxism tend to exhibit higher levels of depression compared to those without [14]. Yet, it remains unclear whether the observed TMD originates from abnormal inflammatory responses or from parafunctional habits influenced by depressive states.

Currently, bruxism detection can be accomplished through both conventional and digital impressions. A comparative in vitro study evaluating three-dimensional (3D) analysis accuracy demonstrated that digital impressions of the full arch are a reliable alternative to conventional methods [15]. With advancements in digital dentistry, 3D analysis has also shown promise in assessing occlusal tooth wear, especially for the clinical evaluation of posterior tooth wear, where it has proven to be a reliable tool for comparison [16,17]. This advancement in dental 3D scanning sheds light on the application of detecting bruxism in both human and animal studies.

The complex interplay between stress-induced depression and bruxism continues to be a topic of debate in the scientific community. Human studies face inherent limitations due to uncontrollable factors, making it challenging to draw definitive conclusions. To address these gaps in our understanding, a study using an animal model was conducted to investigate the relationship between depression and bruxism by examining the wear patterns of lower molars in these rats. This approach allows us to control variables that are difficult to manage in human studies, potentially offering new insights into the mechanisms linking mental health and oral health.

The objective of this study is to analyze the impact of stress-induced depression on the dental microstructure of rats to uncover potential indicators that could serve clinical applications, and we hypothesize that this will result in significant microstructural alterations in the lower molars of rats, which can be detected and quantified using advanced 3D dental scanning techniques.

## 2. Materials and Methods

### 2.1. Animal Model

**Figure 1:**
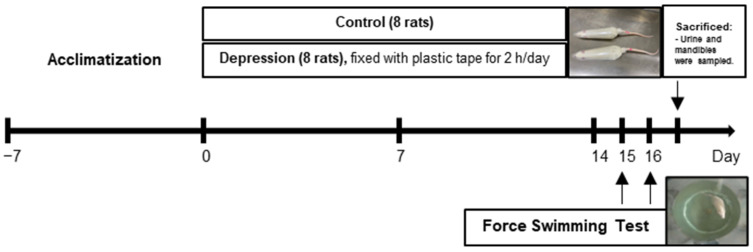
Diagram of the experimental design. After a 7-day acclimatization period, rats were randomized and assigned to the depression group, which was subjected to restraint stress (2 h/day) for 2 weeks. Depression-like behavior was evaluated using the Forced Swim Test (FST) on days 15 and 16. Following behavioral assessments, rats were sacrificed, and tissue samples were collected for analysis.

The animal protocols employed in this study were approved by the Institutional Animal Care and Use Committees of Thammasat University and Khon Kaen University. All experimental procedures adhered to the guidelines set forth in the National Research Council’s Guide for the Care and Use of Laboratory Animals, and they were conducted in full compliance with relevant ethical standards for animal research (IACUC-KKU-39/63). The experimental timeline is shown in Figure 1.

### 2.2. Body Weight, Food, and Water Measurements

Weight measurements were recorded daily using a digital weighing scale for each rat to monitor changes over the 14-day period. Weekly, each rat was placed in metabolic cages for 24 h. At the end of this period, the remaining food and water were collected and weighed. The amount of food and water consumed was determined by subtracting the remaining quantities from the initial amounts provided.

### 2.3. Stress-Induced Depression

Sixteen male Wistar rats, 8 weeks old and weighing 180 to 200 g, were purchased from Nomura Siam International (Bangkok, Thailand) and used in this study, which was sufficient to observe significant differences in microstructural changes due to stress-induced depression from our previous study [11]. The rats were randomly divided into 2 groups (n = 8 in each group), including a control group and a restraint-stress-induced depression group. Each rat was subjected to being immobilized in a 24 × 6 cm transparent plastic cylinder fixed with plastic tape for 2 h/day, 7 days/week for 2 weeks based on our previous study [11,18]. Briefly, a 1 cm hole was made at the end of the cylinder for breathing, and the restraint stress procedure was performed daily and separately from the controls in a quiet room to avoid any confounding factors. On days 15 and 16, the behavioral test was performed to evaluate pre-training and examine depressive-like behaviors using the forced swimming test. All rats were fed standard chow rodent food and had free access to the diet and distilled water. At the end of the study, all rats were euthanized under deep anesthesia using 5% isoflurane [5,11,19]. The adrenal glands were weighed, and skull samples were collected. The skulls, including the mandible with all lower molars, as well as the adrenal glands, were fixed in 4% paraformaldehyde. The lower jaws with molars were preserved at 4 °C for 3D scanning, and we acquired data for cusp wear analysis [20].

### 2.4. Forced Swim Test (FST)

The FST is a hallmark of depressive-like behavior in rodents and consists of training and testing sessions on two consecutive days. The rats had an initial 15 min swimming session for training purposes with no data collection. After the first 15 min swim sessions on day 15, the rats were removed from the cylinders and dried with towels before being returned to their cages. On day 16, a 5 min test was performed, and the rats’ behavior was digitally recorded. The FST was conducted by placing rats in individual glass cylinders (45 cm tall and 20 cm in diameter) containing 25 °C tap water [11]. In the experiments, infrared video cameras were used to record post-recording measurements of the number and duration of swimming behaviors, climbing, immobility behaviors, and fecal pellets. An increase in immobility duration and fecal pellet count indicated heightened depression-like behaviors and stress responses in rats [21,22].

### 2.5. Urinary Corticosterone Assay

After the final stress induction session on day 14, the rats were placed in metabolic cages (model 3700M071-01CS; Tecniplast, Varese, Italy) for 12 h (Day 15) to collect urine prior to undergoing the FST on day 16. The assessment of urine corticosterone (CORH) can be a valid and sensitive biomarker for both physiological and pharmacologically induced stress [22,23,24]. The urine samples were then centrifuged at 2500 rpm for 10 min. After centrifugation, the urine was isolated and stored at −20 °C for subsequent CORH analysis using a commercially available enzyme immunoassay kit (Immunodiagnostic Systems Ltd., Tyne and Wear, UK). All serum samples were analyzed in duplicate, and mean values were calculated.

### 2.6. Adrenal Preparation and Histological Analysis

At the end of the study, the adrenal glands were cleaned, and fat adhesions were removed. After that, they were weighed (mg) relative to body weight (g), as per the previous study [25]. Adrenal glands were dissected, fixed in 4% paraformaldehyde, and processed for histological analysis. The tissue was embedded in paraffin, sectioned into 5 μm thickness, and stained with Hematoxylin and Eosin (H&E). H&E staining highlights the structural components of the adrenal gland, including the cortex and the medulla [26]. The cortex is divided into three zones, with the zona fasciculata being responsible for corticosterone production. Histological analysis involved measuring the thickness of the cortex and the medulla, as well as the zona fasciculata, by examining the distinctive cellular arrangement under the light microscope (Nikon eclipse Ts2-Fl, BX, Olympus, Tokyo, Japan. The thickness of the zona fasciculata can be an indicator of corticosterone production in stress responses, with hypertrophy suggesting increased hormone synthesis. Quantitative measurements of these zones were performed using NIS-Element BR version 4.0 (Nikon, Tokyo, Japan) image analysis software to assess structural changes, which may correlate with the physiological response to stress or other experimental conditions.

### 2.7. 3D Model Acquisition

The bilateral mandibles were collected and stabilized in the sample holder for 3D scanning. The lower molars and the surrounding mandibular bone were scanned at the end of the stress-induced depression experiment using a laboratory 3D surface scanner by structured light/Blue LED Multiline (Dental laboratory 3D scanner E2, 3Shape, Copenhagen, Denmark) to obtain the highest quality of anatomical shape of all molars for the 3D Standard Tessellation Language (STL) models. The digitized 3D models were thereafter imported and analyzed through 3D volume analysis and matched objects for M1 cusp point measurement.

### 2.8. The 3D Cusps’ Morphologic Analysis

The scanning of each sample was performed with a mean accuracy of 10 μm. The scan projections were normalized and used in object reconstruction (Autodesk Meshmixer software version 3.5, San Francisco, CA, USA) to obtain the best matched size. The comparison between the 2 samples was matched together, and the resulting objects of the samples were presented and visualized in 3D by using the 3D compare function in Geomagic Control X software version 2022.1.0.70 (Australian National University, Canberra, Australia). Then, the volume of the cusp tips from each 3D model was assessed using the “Measure Volume” function. The volume measurements for each model were then exported as numerical data for further statistical evaluation. Therefore, the parameters, such as the cusp tip landmarks, were compared to analyze the position of cusp tips by using the “Local Average Tagging” function. The 3D difference of each point, as referenced from the cusp tip landmarks, was exported as numerical data for subsequent statistical analysis.

### 2.9. Scanning Electron Microscopy (SEM)

The molars with attached mandibles were disinfected with 0.5% sodium hypochlorite. The microstructure was first visualized using a microscope. Then, these samples were processed through air drying, mounted on standard SEM stubs using a conductive adhesive, and gold coated (Emitech K-250 sputter coater, Quorum Technologies, Lewes, East Sussex, UK). All of the specimens were then observed on an FEI XL30 FEG (FEI, Eindhoven, The Netherlands) high-resolution SEM fitted with an Everhart–Thornley detector for secondary electrons and a coaxial detector for backscattered electrons. All micrographs were directly obtained in digital form as 1424 by 968 pixel, 8 bpp TIFF files. For each lesion, the location and shape of the defect and the microwear features of the enamel and dentin were analyzed and photographed at a magnification ranging from 25× to 5000×. Data analysis involved comparing two main groups—the control and depression groups—separated by side (left and right), resulting in four sub-groups: right depression, left depression, right control, and left control (n = 8 each).

### 2.10. Statistical Analysis

All cusp point difference data are presented as the mean ± standard deviation (S.D.), as indicated in the figure legends and tables. Statistical analyses were performed using SPSS version 23 software (SPSS Inc., Chicago, IL, USA). The procedures for quantifying parameters from the 3D objects’ analysis were all performed in triplicate in a blinded fashion with no knowledge of the groups. Differences in cusp volume among different rats were tested in a paired manner through t-tests after the normal distribution was measured using the Shapiro–Wilk test. Potential effects of cusp point differences were explored through visual inspection of relevant Box and Whisker plot and statistical analysis of the difference between two means divided by a standard deviation using Cohen’s d. The effect size, calculated using Cohen’s d, was 0.3–0.8. The data were double-checked by another investigator (PP). All data acquisition and analysis were completed blindly, and each experiment was performed by different observers independently (PT, PJ, CL). Results with *p* < 0.05 were considered significant.

## 3. Results

### 3.1. Physical and Behavioral Responses in Stress-Induced Depression

**Figure 2:**
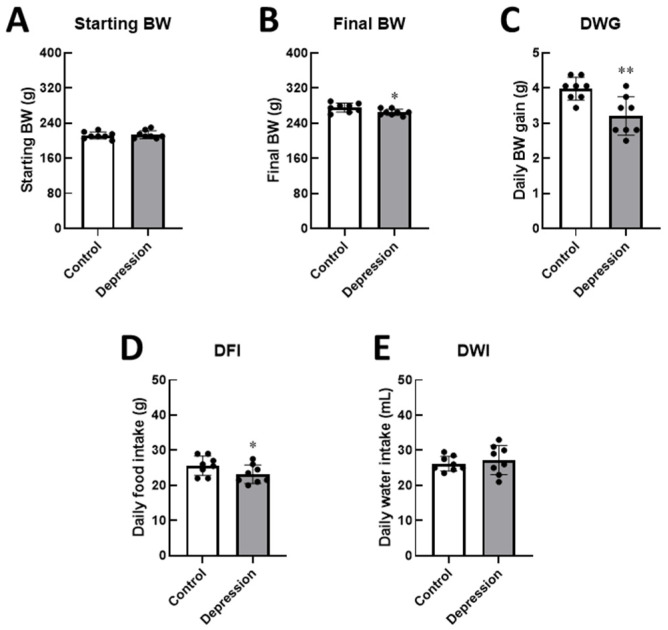
Physical changes and eating behaviors in control and stress-induced depression rats. Changes in the levels of (**A**) staring and (**B**) final body weight (BW), (**C**) daily weigh gain (DWG), (**D**) daily food intake (DFI), and (**E**) daily water intake (DWI) were assessed and compared between the two groups. (* *p* < 0.05, ** *p* < 0.01).

At the start of the study, there were no differences in body weight between the groups (211.90 ± 7.99 g vs. 213.80 ± 9.16 g). However, after 2 h per day of stress exposure for 14 days, rats in the depression group exhibited a lower final body weight compared to the control group (275.60 ± 10.16 vs. 265.00 ± 7.07 g). In the depression group, restraint stress significantly decreased daily body weight gain (3.96 ± 0.32 g vs. 3.20 ± 0.55 g, *p* < 0.01) and daily food intake accordingly (25.63 ± 2.74 g vs. 23.19 ± 2.6523.19 g, *p* < 0.05), as shown in Figure 2 and Table S1. These results indicate the impact of stress on feeding behaviors, suppressing eating behavior and affecting physical growth in stressed male rats. As expected, stress induction reduced daily food intake, while water intake slightly increased in stressed rats (26.19 ± 2.10 mL vs. 27.25 ± 4.17 mL), possibly as a compensatory response.

**Figure 3:**
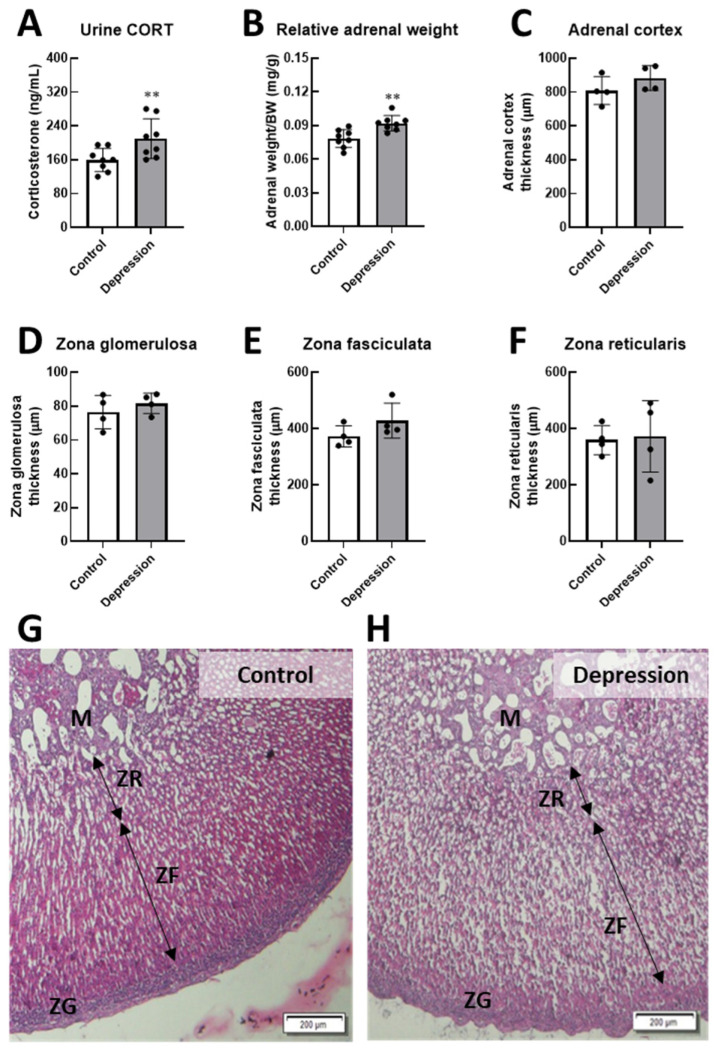
Hormonal and adrenal structural responses in control and stress-induced depression rats. (**A**) Changes in urinary corticosterone (urine CORT) levels, (**B**) the relative wet weight of the adrenal gland to body weight, and histological features of adrenal gland layers—(**C**) adrenal subregion cortex, (**D**) zona glomerulosa (ZG), (**E**) zona fasciculata (ZF), (**F**) zona reticularis (ZR), and the medulla (M)—were assessed and compared between groups. Photograph of histological adrenal gland sections stained with H&E of the control group (**G**) compared to depression group (**H**). (** *p* < 0.01).

Furthermore, the wet adrenal gland weight relative to final body weight was significantly increased (0.08 ± 0.008 mg/g vs. 0.09 ± 0.007 mg/g, *p* < 0.01), possibly due to rich vascularization and well-functioning adrenal tissues supporting corticosterone synthesis and release. Urinary CORT levels in the depression group were elevated (159.30 ± 27.44 ng/mL vs. 209.80 ± 46.94 ng/mL, *p* < 0.01). However, adrenal gland thickness showed a tendency to increase, particularly in the zona fasciculata (372.60 ± 37.29 μm vs. 428.80 ± 61.91 μm, *p* = 0.085), possibly due to an increase in adrenal cell volume, which may contribute to the overall thickening of the adrenal cortex (Figure 3).

**Figure 4:**
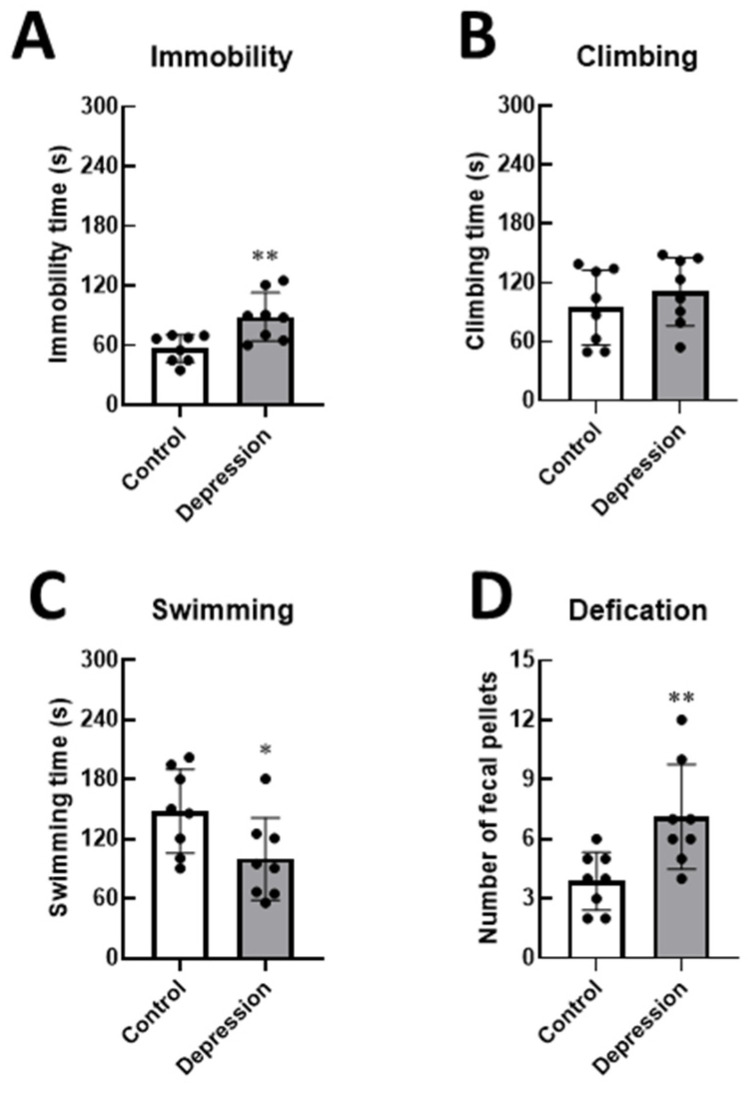
Changes in the level of depression-like behaviors in stressed male rats were assessed using the forced swimming test (FST) by recording time spent in immobility (**A**), climbing (**B**), and swimming (**C**), as well as the number of fecal pellets (**D**) in control and stress-induced depression rats (* *p* < 0.05, ** *p* < 0.01).

Additionally, stressed rats showed increased FST immobility (56.94 ± 13.78 s vs. 88.91 ± 24.17 s, *p* < 0.01) and a significant reduction in FST swimming (148.20 ± 42.23s vs. 100.00 ± 41.35 s, *p* < 0.01), suggesting that this group of rats exhibited depression-like behaviors without any changes in climbing activity (Figure 4). Furthermore, the number of fecal pellets was higher in depressed rats, indicating a heightened stress response compared to the control group (*p* < 0.01). These results indicate that immobilization for 2 h/day over 14 days induced depression-like behaviors and exacerbated the consequences of stress-induced depression in male rats.

### 3.2. 3D Tooth Wear Analysis

**Figure 5:**
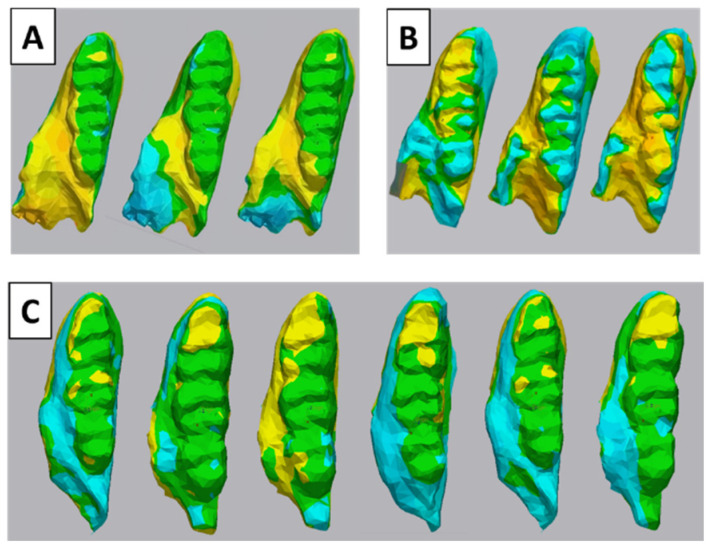
Representative illustration of structural comparisons of lower molars. (**A**) Control–control, (**B**) depression–depression, and (**C**) control–depression. Green indicates slight to no difference between the two samples (*p* < 0.05 mm), while yellow and blue indicate a difference greater than 0.05 mm.

The lower molar results after the best-fit merging of two objects showed that the non-difference area (green color, difference < 0.2 mm) is more extensive, covering almost all of the tooth’s surface, suggesting that the control group has less variation of cusp change than the depression group (Figure 5A,B). Nevertheless, the molar surface compared between the control group and depression group was changed, especially on the cusp tip area (yellow color) (Figure 5C).

**Figure 6:**
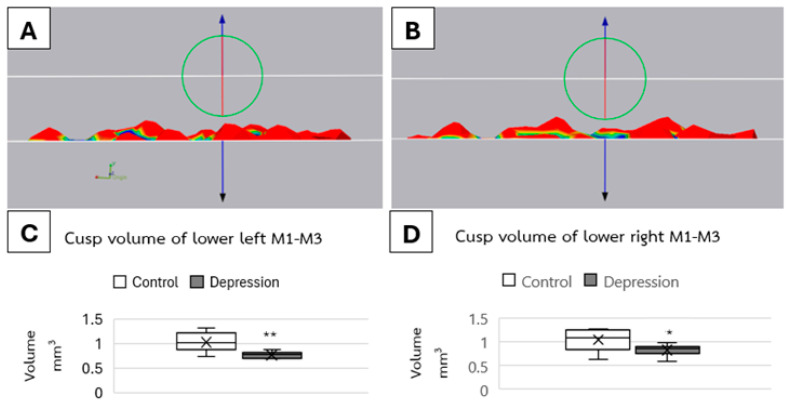
Representative 3D segmentation of cusp volumes in the first to third molars (M1–M3). Comparison of cusp morphology between the (**A**) control group and (**B**) depression group. Quantitative analysis of mean cusp volumes for the (**C**) left and (**D**) right sides. (* *p* < 0.05, ** *p* < 0.01).

The 3D cusp volume analysis showed that there were significant differences in mean total cusp volume between groups on both the left (*p* < 0.01) and right sides (*p* < 0.05), as shown in Figure 6. On the left side, the volume of molars was 1.03 ± 0.12 mm^3^ in the control group and 0.78 ± 0.11 mm^3^ in the depression group. The right side also showed the same trend of difference, which was 1.04 ± 0.11 mm^3^ in the control group and 0.83 ± 0.08 mm^3^ in the depression group.

**Figure 7:**
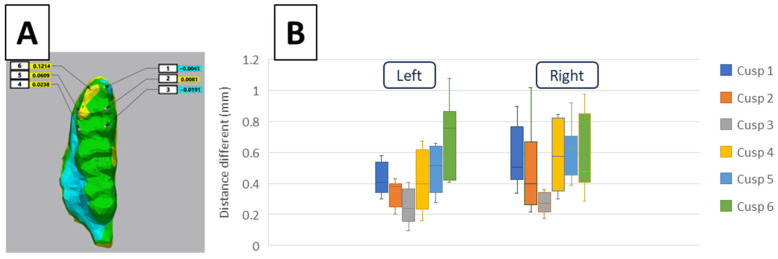
Representative illustration of morphological analysis of the left first molar (M1). The six landmark points of the left M1 (**A**), which were used to calculate the distance differences. The distance difference analysis of the cusp of the M1 shows the difference between 2 groups on the left side (Left) and right side (Right) (**B**).

The distance difference analysis of six landmark points merging between two groups one by one on every rat (Figure 7A) revealed that there were statistically significant differences among the distance differences in six landmarks (*p* < 0.001) (Figure 7B). Specifically, the largest differences were observed between cusps three and six on the left side and cusps three and five on the right side, with Cohen’s d values reflecting large effect sizes of 0.8 and 0.7, respectively.

### 3.3. Microscopic Analysis

**Figure 8:**
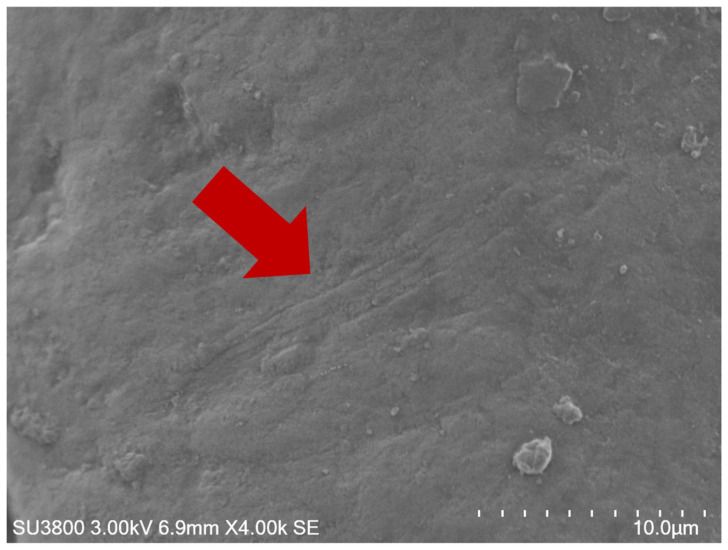
The nanostructure of the cusp tip surface revealed scratch lines in the depression group (red arrow).

The cusp areas, suspected to be subjected to wearing forces, were further investigated using SEM. In the macroscopic view, the overall cusps appeared quite similar between the two groups. Some areas of the cusps in the depression group appeared slightly more polished than those in the control group. Interestingly, in the depression group, scratch lines were particularly evident at cusps five and six (Figure 8).

## 4. Discussion

By exploring these connections in a controlled environment, we aim to determine the microstructural changes of lower molars in rats exposed to stress-induced depression by using a method of 3D analysis via a dental scanner. Our findings potentially inform future strategies for diagnosis, prevention, monitoring, and treatment of depression and TMD in clinical settings. Depression is a multifaceted psychiatric disorder that affects millions of individuals worldwide. The complexity of depression, compounded by its heterogeneous nature, necessitates a robust modeling approach to accurately reflect human pathology. Understanding its underlying mechanisms is crucial for holistic healthcare. Animal models, particularly rodent models, have been extensively used to study depression due to ethical and practical limitations in human research. The Wistar rat strain exhibits hypersensitivity to stress and displays depression-like behaviors, such as lower food consumption, elevated adrenal gland weight, increased levels of serum and urinary corticosterone, and reduced nocturnal activity, similar to those observed in humans, suggesting that it could serve as a valuable model [6,11,18], making it a useful model for translational studies and novel intervention development and paralleling findings in human depression. Hence, the utility of rat models in studying the neurobiological aspects of stress and depression is documented [24,27]. While ethical constraints limit direct investigations in humans, rodents offer a valuable framework for understanding the underlying mechanisms of these conditions.

Our study found that rats that underwent stress exhibited signs of depression, as indicated by the FST and HPA axis responses, including alterations in adrenal weight and urinary corticosterone levels. The previous study of this stress-induced depression model showed that this group had condylar defects, which represent TMD signs, leading to the exploration of the path to figure out how TMD occurred [11]. Based on recent knowledge, psychological depression possibly leads to the coping of masticatory muscles and bruxism as a means of relieving psychological tension [27,28]. Chewing is an effective behavior for coping with anxiety via the existing pathway [29], plus stress can be altered by the activity of the HPA axis and the autonomic nervous system [30]. The mechano-electrochemical signals inspired our study to further investigate the remnants of mechanical mechanisms that extend to TMD causes [29]. For example, the force of occlusion by anterior elevation and sustained joint loading possibly cause TMD [31]. Hence, investigating tooth wear could explain TMD lesions caused by overuse of chewing muscles.

Recent clinical studies investigating the association between tooth wear and bruxism remain inconclusive [32,33,34]. Based on the findings from human and animal studies, the mechanism of sleep bruxism is still uncertain [35]. The higher prevalence and increased severity of tooth wear among male adolescents may be influenced by steroid hormones [36]. Animal models offer a more controlled environment for exploring the causes of TMD, allowing for the regulation of variables like diet, the environment, and the hormone corticosteroid. Previous research has demonstrated that stress-induced bruxism can lead to physiological effects, including gastric ulcers [37]. Additionally, stress exposure in rats has been linked to heightened mechanical sensitivity of the joint [38], and low occlusal loading in rats has been shown to impair neuromuscular behavioral development [39]. However, measuring masticatory muscle activity in rats may not fully represent the psychological aspects of depression in humans, as the equipment is invasively attached to animals. Indirect analysis of murine molars is a viable approach, as these teeth are more comparable to human molars than murine incisors, which are capable of continuous regeneration [40]. Our findings suggest that stress-induced depression, which could contribute to bruxism and TMD referring to the epidemiological data [12], may serve as a novel model for studying bruxism through observed tooth wear.

In our study, we applied this method to investigate differences between two groups of rats, focusing on total cusp volume and the cusp height, which showed greater variation on the right side. The observed molar cusp loss in the depression group suggests that tooth wear, potentially linked to bruxism-like behavior, occurred in these animals [15,16,17]. The preferred chewing side in rats has been explored through various studies, revealing a tendency towards right side preference, with 72.7% of rats exhibiting a right paw preference [41]. This preference for the right side may be linked to lateralization in brain function, similar to handedness in humans [42]. A significant majority of individuals exhibit a preferred chewing side, with studies showing that over 90% of subjects favor unilateral chewing [43]. This preference can lead to uneven wear on teeth, as the side used more frequently experiences greater stress and wear. Chewing side preference is associated with structural changes in the temporomandibular joint (TMJ), including altered condylar shape morphology and position [44,45]. Habitual unilateral chewing may influence dental health and facial symmetry, leading to asymmetries in the TMJ and facial structure [46]. Previous studies have found that TMD presents more severe defects on the left side [11], indicating that our findings of tooth wear may be a leading cause of TMD.

In the depression group, SEM observations revealed scratch lines at the cusp tips. Vertical scratches on tooth wear facets are significant indicators of the mechanical and functional aspects of dental wear. This phenomenon is influenced by various factors in humans, including masticatory patterns, dietary habits, and the interplay of different wear mechanisms. Tooth grinding contributes to the development of vertical scratches, indicating excessive wear [47]. In cases of tooth attrition in humans, the enamel surface appears smooth and polished with stripes and striations compatible with protrusion and lateral occlusal movements microscopically [48]. Identifying vertical scratches can aid in assessing the risk of further tooth wear and guide preventive measures.

Additionally, a narrative overview study suggested that sleep bruxism is associated with tooth wear [49]. Currently, monitoring the progression of tooth wear clinically is possible using an intraoral scanner. The study by Diaz-Flores showed promising tooth wear detection using a full-arch dental scan after 1 year [50]. Monitoring tooth wear for 24 and 36 months showed that the buccal load-bearing cusps had lost value among participants [51,52]. Recent advancements have enabled the monitoring of tooth wear progression using artificial intelligence (AI) integrated with intraoral scanners [53]. This technological breakthrough translates bench side research into bedside applications, illuminating new avenues for assessing and managing tooth wear in clinical practice.

While our study identified distinct molar tooth wear in rats with stress-induced depression, certain limitations should be acknowledged. Differences in anatomical jaw movement and anatomical cusp between clinical and pre-clinical models [54], as well as the complexity of depression in real-life situations, pose challenges for direct comparisons. Our findings suggest that bruxism may play a role in the development of TMD; however, this interpretation should be approached with caution, as we did not investigate immune system involvement. Despite these limitations, the non-invasive, indirect method we employed effectively captured parafunctional behavior without impacting the rats during experimentation. Future studies could benefit from testing the surface hardness to strengthen the reliability of dental tissue characteristics and using continuous video monitoring incorporated with AI to track jaw movements over 24 h, a technique applicable to both clinical and pre-clinical research. This advancement would enhance our understanding of sleep and awake bruxism associated with depression. While advancements in treatments for depression, such as transcranial magnetic stimulation [55], show promising results, our findings contribute to preventive strategies aimed at reducing the occurrence of TMD and highlight the way for monitoring depressive symptoms through a dental scanning perspective.

## 5. Conclusions

In conclusion, this study demonstrates that stress-induced depression over 14 days in male rats presents characteristics and symptoms of depression, with physical, biochemical, and behavioral changes. The deteriorative effects of depression are also associated with significant molar tooth wear, suggesting a potential link between bruxism-like behavior and the development of TMD.

## Data Availability

The original contributions presented in this study are included in the article. Further inquiries can be directed to the corresponding authors.

## Supplementary Materials

The following supporting information can be downloaded at https://www.mdpi.com/article/10.3390/life15050712/s1, Table S1: Summary of all parameters assessed in the present study (Figure 2, Figure 3, Figure 4, Figure 5 and Figure 6).
