# Supplementary material for: Stress-Induced Depression and Its Effects on Tooth Wear in Rats: A 3D Dental Scan Imaging Perspective
Source: Life (Basel). 2025 Apr 28;15(5):712. doi: 10.3390/life15050712 (PMC12113425; doi:10.3390/life15050712)
Supplement: Supplementary file 1 [file life-15-00712-s001.zip › life-3618373-supplementary.pdf]

Supplementary Table

Stress-Induced Depression and Its Effects on Tooth Wear in Rats: A 3D Dental Scan Imaging Perspective

Preeyarat Plongniras[1], Sarawut Lapmanee[2], Natchayaporn Thonapan[3], Phuripong Thangsombat[4], Phongsakorn Janthaphim[4], Chanakarn Lertkarnvijai[4], Pattama Chailertvanitkul[5], Supawich Morkmued[4,*]

[1]Department of Prosthodontics, Faculty of Dentistry, Khon Kaen University, Khon Kaen, Thailand 1; preepl@kku.ac.th
[2]Chulabhorn International College of Medicine, Thammasat University, Pathum Thani, Thailand 2; lapmanee@tu.ac.th
[3]Department of radiological technology, Faculty of Allied Health Sciences, Thammasat University, Pathum Thani, Thailand 3; natchayaporn.t@allied.tu.ac.th
[4]Department of Preventive Dentistry, Pediatric Dentistry Division, Faculty of Dentistry, Khon Kaen University, Khon Kaen, Thailand 1; supamo@kku.ac.th
[5]Department of Restorative Dentistry, Faculty of Dentistry, Khon Kaen University, Khon Kaen, Thailand 1; patchai@kku.ac.th

*Correspondence: supamo@kku.ac.th

Supplementary Table S1. Summary of all parameters assessed in the present study (Figure 2 to 6)

**Parameter**	**Control group (95% CI of mean)**	**Depression group (95% CI of mean)**	***p*-value**
Starting body weight (g)	211.90 ± 7.99 (205.20 to 218.60)	213.80 ± 9.16 (206.10 to 221.40)	0.034
Final body weight (g)	275.60 ± 10.16 (267.10 to 284.10)	265.00 ± 7.07 (259.10 to 270.90)	0.015*
Daily weight gain (g)	3.96 ± 0.32 (3.71 to 4.26)	3.20 ± 0.55 (2.74 to 3.66)	0.002**
Daily food intake (g)	25.63 ± 2.74 (23.34 to 27.91)	23.19 ± 2.65 (20.98 to 25.40)	0.046*
Daily water intake (mL)	26.19 ± 2.10 (24.43 to 27.95)	27.25 ± 4.17 (23.77 to 30.73)	0.265
Urine corticosterone (ng/mL)	159.30 ± 27.44 (136.30 to 182.20)	209.80 ± 46.94 (170.50 to 249.00)	0.010**
Relative adrenal weight/ body weight (mg/g)	0.08 ± 0.008 (0.072 to 0.085)	0.09 ± 0.007 (0.086 to 0.098)	0.001**
Adrenal cortex thickness (µm)	808.60 ± 82.53 (677.20 to 939.90)	883.30 ± 73.72 (766.00 to 1,001.00	0.113
Zona glomerulosa thickness (µm)	76.47 ± 9.94 (60.65 to 92.28)	81.71 ± 6.02 (72.13 to 91.28)	0.201
Zona fasciculate thickness (µm)	372.60 ± 37.29 (313.30 to 431.90)	428.80 ± 61.91 (330.30 to 527.30)	0.085
Zona reticularis thickness (µm)	359.50 ± 51.78 (277.10 to 441.90)	372.80 ± 126.3 (171.80 to 573.80)	0.421
Immobility time (s)	56.94 ± 13.78 (45.42 to 68.46)	88.91 ± 24.17 (68.70 to 109.10)	0.003**
Climbing time (s)	94.89 ± 38.02 (63.11 to 126.70)	111.10 ± 34.57 (82.15 to 140.00)	0.194
Swimming time (s)	148.20 ± 42.23 (112.90 to 183.50)	100.00 ± 41.35 (65.47 to 134.60)	0.019*
Fecal pellet numbers	3.88 ± 1.46 (2.66 to 5.09)	7.13 ± 2.64 (4.92 to 9.33)	0.004**
Left cusp volumes in the first to third molars (mm^3)	1.03 ± 0.12 (0.87 to 1.20)	0.78± 0.11 (0.72 to 0.83)	0.002**
Right cusp volumes in the first to third molars (mm^3)	1.04 ± 0.11 (0.84 to 1.23)	0.83± 0.08 (0.73 to 0.92)	0.018*

Data were presented as mean ± standard deviation (SD) with 95% Confidence Interval (lower to upper CI)
